# Comparison of Statistical Models for Analyzing Wheat Yield Time Series

**DOI:** 10.1371/journal.pone.0078615

**Published:** 2013-10-29

**Authors:** Lucie Michel, David Makowski

**Affiliations:** 1 Institut National de Recherche Agronomique, Unité Mixte de Recherche 211 Agronomie, Thiverval-Grignon, France; 2 AgroParisTech, Unité Mixte de Recherche 211 Agronomie, Thiverval-Grignon, France; Pacific Climate Impacts Consortium, Canada

## Abstract

The world's population is predicted to exceed nine billion by 2050 and there is increasing concern about the capability of agriculture to feed such a large population. Foresight studies on food security are frequently based on crop yield trends estimated from yield time series provided by national and regional statistical agencies. Various types of statistical models have been proposed for the analysis of yield time series, but the predictive performances of these models have not yet been evaluated in detail. In this study, we present eight statistical models for analyzing yield time series and compare their ability to predict wheat yield at the national and regional scales, using data provided by the Food and Agriculture Organization of the United Nations and by the French Ministry of Agriculture. The Holt-Winters and dynamic linear models performed equally well, giving the most accurate predictions of wheat yield. However, dynamic linear models have two advantages over Holt-Winters models: they can be used to reconstruct past yield trends retrospectively and to analyze uncertainty. The results obtained with dynamic linear models indicated a stagnation of wheat yields in many countries, but the estimated rate of increase of wheat yield remained above 0.06 t ha^−1^ year^−1^ in several countries in Europe, Asia, Africa and America, and the estimated values were highly uncertain for several major wheat producing countries. The rate of yield increase differed considerably between French regions, suggesting that efforts to identify the main causes of yield stagnation should focus on a subnational scale.

## Introduction

Agriculture will be faced with major challenges in the next few decades. The number of undernourished people was estimated at 868 million for the period 2010−2012 [1], indicating that food demand has not yet been satisfied in some parts of the world. The situation may worsen in the near future due to i) current demographic trends, with the world population likely to reach 9.3 billion by 2050 [2], ii) increasing consumption of meat products in several major developing countries, iii) the development of biofuels, iv) limited possibilities for increasing the cultivable area [2], [4].

Several studies have recently shown that, after a period of strong yield increase, yield levels are currently stagnating in several countries. The average yield of cereal crops increased by more than 98% worldwide, and by more than 187% in France from 1960 to 1990 [5]. There were several reasons for this positive trend: genetic improvement of crop cultivars, increase in the use of chemical inputs (fertilizers, insecticides, herbicides and fungicides), mechanization, and irrigation. These improvements led to an approximately linear increase in crop yields in many countries. However, since the 1990s, yield increases for several major cereal crops (wheat, maize, rice, barley or oat) have slowed down. In some countries, yield levels have remained constant or have even declined for some crops [3], [4], . This is the case for wheat, for which several authors have recently shown much slower rates of yield increase than the period prior to 1990s, with yield stagnation in several countries, including France [3], [8], [14] and Switzerland [6]. These results have raised significant concerns in the scientific community about the ability of agriculture to feed the world in the future.

Statistical analyses play a key role in current research studies on food security [14], [15] where yield time series analysis is used to estimate past yield trends and to predict future yield trends. Various types of statistical models have been used for the analysis of yield time series. Linear regression has been used in many studies [3], [4], [6−8], [11], [13], [14], [16], . Other regression models, such as quadratic regression, bi-linear, tri-linear, and linear-plus-plateau models, have been used in a smaller number of papers. Several authors have shown that quadratic and linear-plus-plateau models tend to perform better in cases of yield stagnation [3], [6−8], [14], .

Statistical methods other than regression models have been used to predict future yield trends. Kumar (2000) [16] compared the performances of linear and quadratic regression models with those of exponential smoothing (also known as the Holt-Winters method) and moving averages. Exponential smoothing has been shown to perform well in a large range of applications [17], [18], but Kumar (2000) [16] showed that the lowest mean square error (MSE) for yield predictions was obtained with the quadratic regression model. However, this result was obtained with a small dataset: yield predictions were assessed for three years at a specific location in Canada. It is, thus, difficult to draw general conclusions from this study.

The dynamic linear model (DLM) is a recently developed statistical method that can be used to estimate past trends and to predict future trends in time series. It has been applied in diverse domains, such as econometrics, signal processing, genetics and population dynamics [19]–[21]. This method is based on the Kalman filter and the Kalman smoother. It is very flexible, because the coefficients of the underlying linear model are adjusted at each time step. Their values are, therefore, not fixed as in classical linear regression; they vary from year to year and could thus account for changes in yield trends (e.g., stagnation, increase or decline in the rate of yield increase). This DLM approach has not yet been applied to analyses of yield time series.

The aim of this study was to compare the performance of eight statistical models, including DLM, for analyzing yield time series and predicting yield trends. We chose wheat as the crop for this analysis, because wheat is an important cereal crop (27% and 58% of total cereal production worldwide and in France, respectively) and because wheat yield time series show a great diversity of trends (increasing, plateauing, decreasing). We used a large number of wheat time series obtained at the national scale (in 120 countries), and at the subnational scale (in 92 French *départements*) to compare the statistical models.

## Materials and Methods

### Data

Three datasets including wheat yield time series were defined for comparing the performances of the statistical methods. They are described below.

**Dataset 1: Global.** The first dataset includes wheat yield time series extracted from the FAOSTAT database [5] for 120 countries. This database has already been used in several studies on yield time series [3], [6], [7], [9], [10]. For most countries, yield time series began in 1961 and ended in 2010. The only exceptions were eight countries created after 1961, for which time series were shorter. In this dataset, bread wheat (*Triticum aestivum L.*) was not distinguished from durum wheat (*Triticum turgidum L.*) and winter wheat was not separated from spring wheat, because no distinction between these crops was made in the FAOSTAT database.

**Dataset 2: France.** The second dataset included winter bread wheat and winter durum wheat yield time series extracted from the AGRESTE database (French Ministry of Agriculture) [22] and from the printed reports of the SSP (*Service de la Statistique et de la Prospective du Ministère en Charge de l’Agriculture*) for 92 (of 96) French *départements* (NUTS-3 level according to the EU nomenclature). The AGRESTE website was used to extract yields from 1989 to 2011 and the SSP reports were used to extract yield data from 1950 to 1988. Four French *départements* were excluded due to a lack of data: Haute-Corse (2B), Corse-du-Sud (2A), Hauts-de-Seine (92) and Paris (75). Note that yield data were available only from 1968 to 2011 for the *départements* located in the Ile-de-France region (Paris area).

**Dataset 3: France (restricted).** The third dataset was a restricted version of Dataset 2 including only the 56 French *départements* for which we were able to fit the linear-plus-plateau model (one of the statistical models tested in this paper, see below). For the other *départements,* the fitting algorithm did not converge toward a solution due to the lack of fit of the linear-plus-plateau model to the data. These convergence problems resulted from the absence of a plateau in the corresponding times series, making it impossible for the algorithm to identify a start date for the plateau. Dataset 3 was used only to compare the linear-plus-plateau model with the other statistical models.

### Models and statistical methods

We assessed the suitability of eight different statistical models for analyzing yield time series. The models were first fitted to the time series included in our datasets and their qualities of fit were compared. The accuracy of the yield predictions obtained with the models was then assessed by cross-validation.

**Model description.** The models considered in this study were: linear, linear-plus-plateau, quadratic, cubic, dynamic linear models (two variants), and Holt-Winters models (two variants).

The linear model (L) has frequently been used to analyze yield trends in previous studies [3], [4], [6−8], [11], [13], [14], [16], . It assumes a constant rate of yield increase over time and is defined as follows:
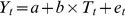
(1)


Where 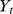 is the yield in year *t*, 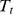 is the year index (with 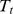 = 1 for the first year of the time series, i.e. for 1950 in Dataset 2 and 3, and for 1961 in Dataset 1), *a* and *b* are the two parameters of the linear trend, and 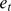 is the residual error equal to the difference between 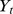 and the linear trend.

The quadratic model (Q) has been used to analyze yield time series in several previous studies [6], [7], [14], [16], [17]. Unlike the linear model, this model does not assume a constant rate of yield increase and is defined as follows: 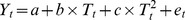
(2)


Where *a*, *b* and *c* are three parameters estimated by fitting the model to the data.

The cubic model (C) is more flexible than Q [14] but includes an additional parameter (*d*) and is defined by: 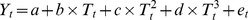
(3)


The linear plus plateau model (LP) is characterized by a marked stagnation [3]. According to this model, yield is assumed to increase at a constant rate *R* before a date 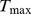 and is then assumed to reach a plateau 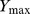:



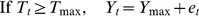
(4)




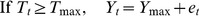
(5)



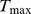
,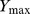, and *R* are three parameters.

In models L, Q and LP, we assume that the residual errors of the model are normally distributed and independent, 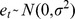. Autocorrelations were estimated as a function of time, to check the assumption of independence for model errors.

Two types of Holt-Winter model [16], [18] were considered in this study. The first model (HWs) was used to predict future yield on the basis of a linear trend. Unlike L, HWs does not assume that the parameter values of the linear trend (intercept and slope) are constant over time, instead assuming that they may vary at each time step. HWs yield predictions are calculated as follows:
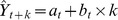
(6)


where 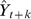
is the *k* year ahead yield prediction (yield prediction *k* years after the last measurement), and 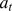 and 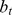 are the two parameters of the linear trend. The values of 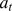 and 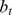 are updated each time a new yield value 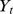 becomes available, as follows:
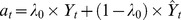
(7)

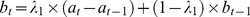
(8)


The algorithm is initialized with 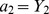 and 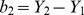. HWs includes only two parameters (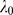
and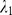) that must be estimated from data, but it can nevertheless accommodate diverse trends characterized by varying increases/decreases in the rate of yield increase over time. The second Holt-Winter model (HW0) considered in this study was a simplified version of HWs that did not include a linear trend (i.e., 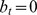). This model is often called “simple exponential smoothing” [16], [18] and is defined by 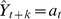 with 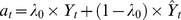. It includes only one parameter 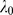 that must be estimated from data. Holt-Winters models generate forecasts of future yield values and are not used to estimate past yield trends.

Two types of dynamic linear model (DLM) were considered [19]–[21]; a polynomial DLM (DLMs) and a random walk DLM (DLM0). The yield predictions of DLMs can also be derived from Eq.(6). However, the values of 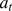 and 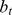 were not calculated with Eqs.(7−8), but with the Kalman smoother algorithm [19], [20]. The parameters 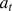 and 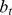 are defined as dynamic random variables and their values are estimated by the conditional expected values of 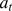 and 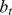 given the available yield data, i.e., 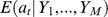 and 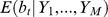 where 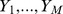 are the *M* yield data of the time series. The Kalman smoother algorithm can also be used to calculate the conditional variances and various quantiles of the conditional probability distributions of 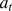 and 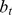 (e.g., 1^st^ and 3^rd^ quartiles). The expected values and variances are calculated analytically with two equations; an observation equation relating yield data to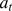, and a system equation describing the changes in 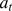 and 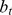 from year to year. The observation equation is defined by
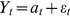
(9)


where 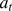 is the yield level at time *t*, and 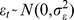. The system equation is defined by 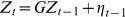
(10)


with 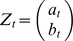, 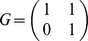, 
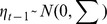
, and 
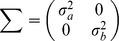


In the DLMs model defined by Eqs.(9−10), the state variables are the time-varying level and slope describing the yield dynamic. These two unobserved state variables are assumed to vary from year to year according to a stochastic process defined by the system equation. The slope 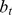 is the local growth rate, the yearly increase of the trend (i.e., the yield increase obtained in one year). The observation equation relates the yield data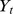, *t* = 1, …, *N*, to the yield level, and the system equation relates the values of the two state variables at time *t* to the values at time *t*-1. The model includes three unknown parameters: 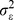, 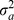, and 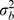. The variance 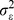 quantifies the variability of yield around the trend. The variances 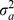, and 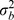 quantify the variability of the level and slope of the yield trend and define their change over time. The three variances 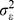, 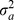, and 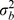 must be estimated from data.

The DLM0 model is a simplified version of DLMs with 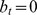 and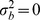. DLM0 corresponds to a random walk model [20] and includes only two parameters that need to be estimated from data.

**Parameter estimation.** Model parameters were estimated for each time series and for each geographical unit (country or French *département*) included in Datasets 1, 2 and 3. The parameters of models L, Q, C, and LP were estimated by ordinary least squares, using the function lm (for L, Q, and C) and nls (for LP) of R software. The model residuals obtained at different dates were not correlated, according to the autocorrelations calculated with the acf function of R. The parameters of the HW0 and HWs models were estimated with the optimizer of the HoltWinters function of R. The parameters of DLM0 and DLMs were estimated by maximum likelihood, with the function dlmMLE of the package dlm of R [19], [20].

**Evaluation.** The models were evaluated in two different ways. First, the goodness-of-fit to past data was assessed for each model by calculating the root mean square error (RMSE), defined as follows: 
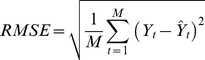
(11)


where 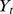 is the yield at time *t*, and 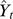 the fitted yield value obtained at the same time with the model adjusted for all available data. RMSE was calculated for each time series from the *M* available yield data (*M* = 49for Dataset 1, *M* = 61 for Dataset 2 and Dataset 3). RMSE values were then averaged over the geographical units (countries or *départements*) of each dataset (Dataset 1, Dataset 2, Dataset 3). RMSE was calculated for each model in turn, with two exceptions, HW0 and HWs, because these two models generate forecasts of future yield values only.

RMSE is useful for evaluating the goodness-of-fit of the models but not for evaluating their prediction errors, because this criterion is calculated by making use of the same data twice, for parameter estimation and for comparing data and model predictions. We evaluated the predictive capabilities of the models, by calculating root mean square error of prediction (RMSEP) for each model in turn. The *k* years ahead RMSEP of the *N* last available data was defined as follows: 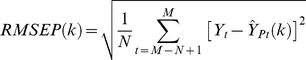
(12)


where 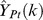 is the yield value predicted at time *t* from the measurements available up to time *t*-*k*. 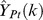 was not calculated in the same way as 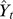. Yield prediction 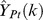 was calculated by fitting the model to the data available before and up to time *t*-*k*. Yield *k* years ahead was then predicted with the fitted model. The value of *k* was set to 1, 2,..., 10 successively, to evaluate short-term and long-term predictions. The procedure was repeated *N* times, by adding one additional data successively.

A RMSEP was calculated for each value of *k,* for Datasets 1 and 2. For Dataset 1, yield was predicted from 1991 to 2010 for each time series, that is *N* = 20. For Dataset 2, yield was predicted from 1991 to 2010 for each time series as well. For example, with Dataset 2 and *k* = 1, yield in 1992 was predicted from all data available up to 1991, yield in 1993 was predicted from all data available up to 1992 and so on. For *k*+5, yield in 1992 was predicted from all data available up to 1987, yield in 1993 was predicted from all data available up to 1988 and so on. It was not possible to calculate RMSEP for the LP model due to the convergence problem.

## Results

### Examples of fitted values

**Figure 1:**
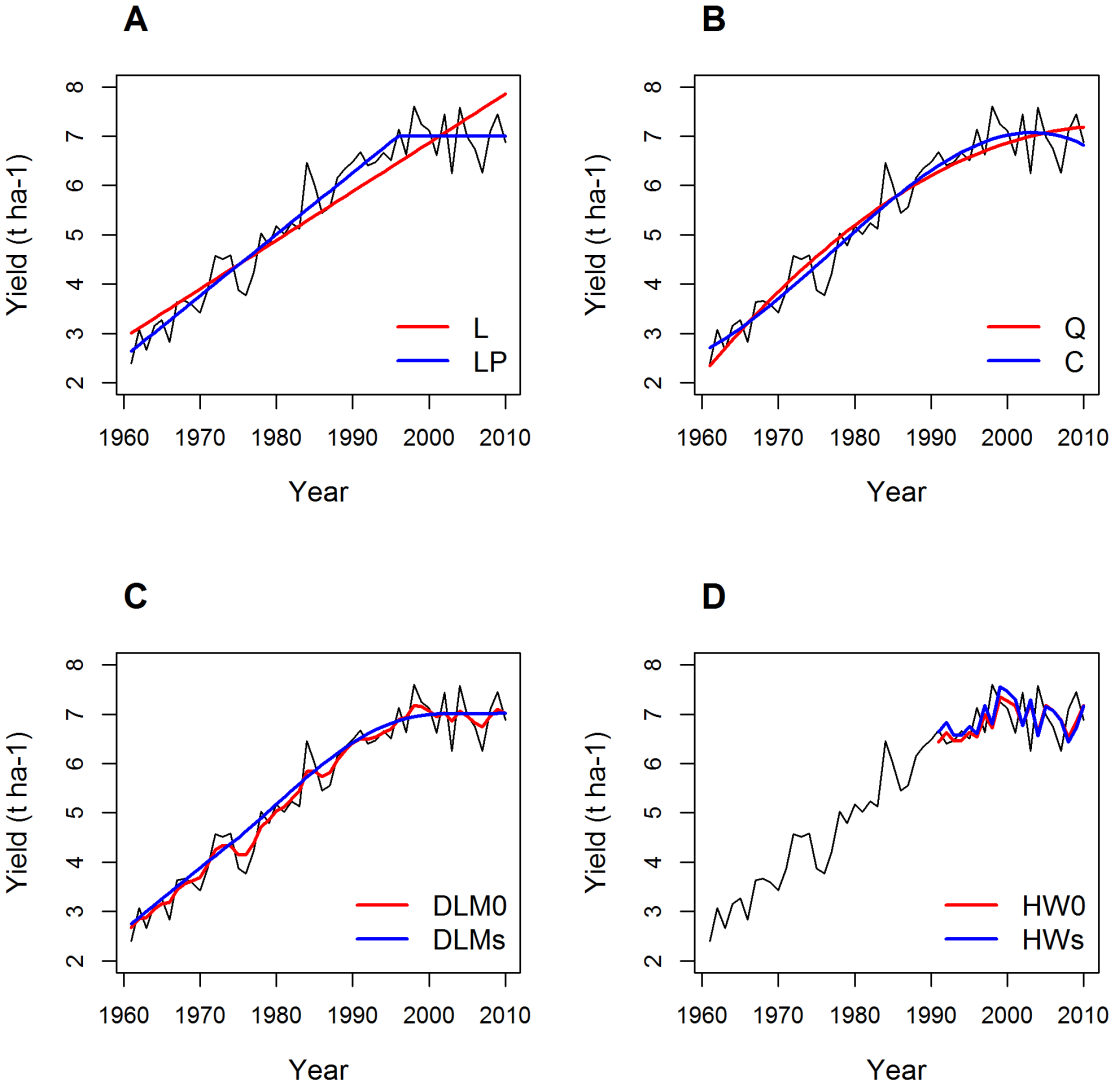
Wheat yield time series in France and fitted values obtained with different models. A: linear (L) and linear-plus-plateau (LP) models. B: Quadratic (Q) and cubic (C) models. C: dynamic linear models with and without trend (DLMs, DLM0). D: Holt-Winters models with and without trend (HWs, HW0).

**Figure 2:**
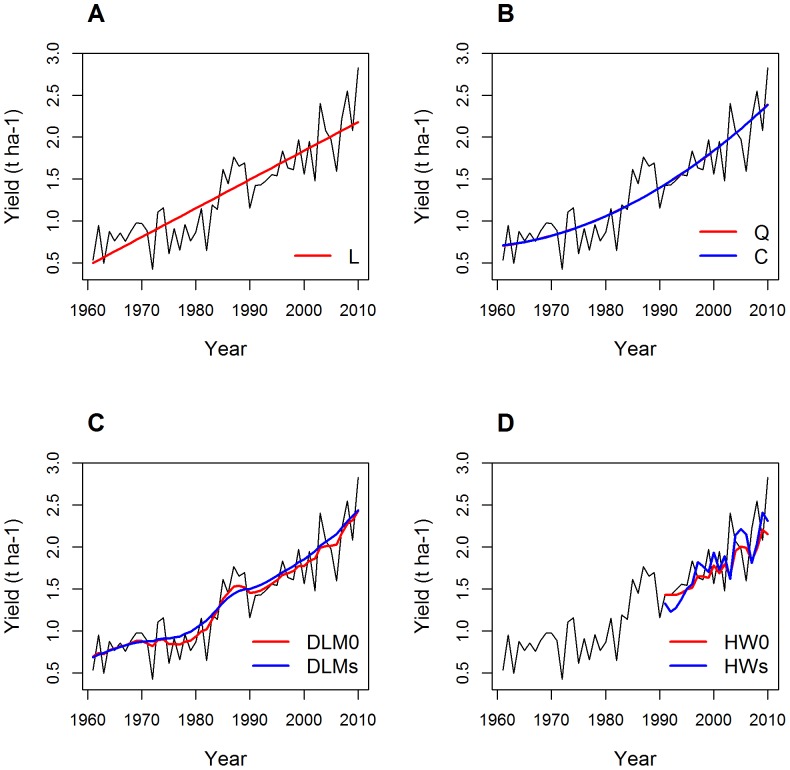
Wheat yield time series in Brazil and fitted values obtained with different models. A: linear (L) and linear-plus-plateau (LP) models. B: Quadratic (Q) and cubic (C) models. C: dynamic linear models with and without trend (DLMs, DLM0). D: Holt-Winters model with and without trend (HWs, HW0).

Examples of wheat yield time series obtained in France and Brazil are shown in Figures 1 and 2, respectively. The two time series shows very different trends. In France, yield was about 2.5 t ha^−1^ in 1961. It strongly increased from 1961 to the mid-1990s, reaching a plateau thereafter at about 6.5 t ha^−1^. Model L did not fit the French data well; yield values were underestimated by this model between 1985 and 2000, and were overestimated before 1975 and after 2000 (Fig.1A). The LP model fitted the data better. Models Q and C also fitted the yield data correctly, but yield was slightly underestimated by Q between 1990 and 2000 (Fig. 1B). DLMs gave a much smoother yield trend than DLM0 (Fig.1C). DLMs also gave a much smoother yield trend than was obtained with LP, with no abrupt change of yield increase rate (Fig.1A, 1C); the yield increase rates obtained with DLMs (i.e., the local slopes of the fitted curve) tended to be constant from 1961 to 1990, gradually decreasing thereafter. Fig.1D presents forecasted yield values obtained with HW0 and HWs for 1991 to 2010. These values are not fitted values, but predicted yields estimated from the previous yield data. The forecasts obtained with HW0 and HWs were very similar for France.

In Brazil, no plateau was observed in wheat yield time series (Fig. 2). Yield was 0.5 t ha^−1^ in 1961, gradually increasing thereafter to reach almost 3 t ha^−1^ in 2010. It was not possible to fit the LP model to this time series due to the absence of a plateau. The L model tended to overestimate yield between 1975 and 1985, and to underestimate yield between 1961 and 1972. Visually, the yield trends fitted by Q and C were almost indistinguishable (Fig. 2B). With both models, the rate of yield increase tended to increase with time, especially after 1990. The yield trends obtained by fitting DLM0 and DLMs were very similar, but that obtained with DLMs was smoother (Fig. 2C). The differences between the yield values forecasted by HW0 and HWs were larger for Brazil than for France; HWs predictions were almost always higher than those obtained with HW0 (Fig. 2D), due to the high rate of yearly yield increase observed in Brazil after 1990. This increasing trend was taken into account by HWs but not by HW0.

### Goodness-of-fit

**Figure 3:**
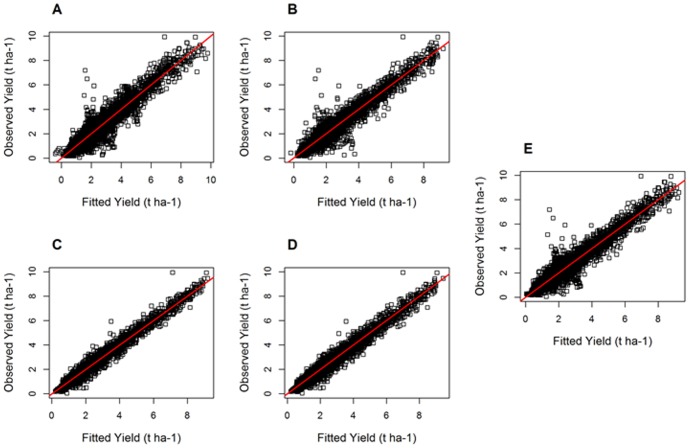
Wheat yield data versus fitted values for all countries and years of the Global dataset (dataset 1). A: Linear model. B: Cubic model. C: DLM0. D: DLMs. E: Quadratic model.

RMSE values are presented for all models and all datasets in Table 1. Yield data and fitted yield values are graphically compared in Figure 3, using Dataset 1 (similar results were obtained with Datasets 2 and 3, not shown). DLM0 was the model with the lowest RMSE values (and thus the best fit) for the three datasets (Table 1). The model with the highest RMSE values (and thus the poorest fit) was the linear model, L. The RMSE values obtained with L were 55 to 97% higher than those obtained with DLM0, depending on the dataset. Thus, DLM0 fitted the data much better than L (Table 1, Fig.3A, 3C). DLM0 also fitted the data better than the models including a larger number of parameters, Q, C, LP, and DLMs (Table 1, Fig. 3). DLMs was the second-best model according to the RMSE values reported in Table 1. This model fitted the data better than the models including the three parameters, i.e., C and LP (Table 1, Fig.3B, 3D). Note that, as mentioned above, it was possible to fit the LP model to data for only a limited number of geographical areas (Dataset 3).

**Table 1:** Root mean square errors (RMSE) obtained with three different datasets for several statistical models: linear regression (L), quadratic regression (Q), cubic regression (C), dynamic linear models with and without trend (DLMs, DLM0), and linear-plus-plateau (LP).

			Models					
Dataset	Quantity	Units	L	Q	C	DLMs	DLM0	LP
France	RMSE	t ha^−1^	0.60	0.54	0.50	0.47	0.38	NA
	Difference^a^	%	55.38	40.85	28.95	22.82	0.00	NA
France (restricted)	RMSE	t ha^−1^	0.59	0.53	0.48	0.46	0.38	0.50
	Difference^a^	%	57.62	42.32	29.13	23.03	0.00	32.30
Global	RMSE	t ha^−1^	0.40	0.35	0.32	0.23	0.20	NA
	Difference^a^	%	96.98	74.03	59.76	15.11	0.00	NA

a The differences with respect to the lowest RMSE values are expressed as a percentage of the lowest RMSE values (RMSE_min_); Difference = 100*(RMSE – RMSE_min_)/RMSE_min_.

### Prediction accuracy

**Figure 4:**
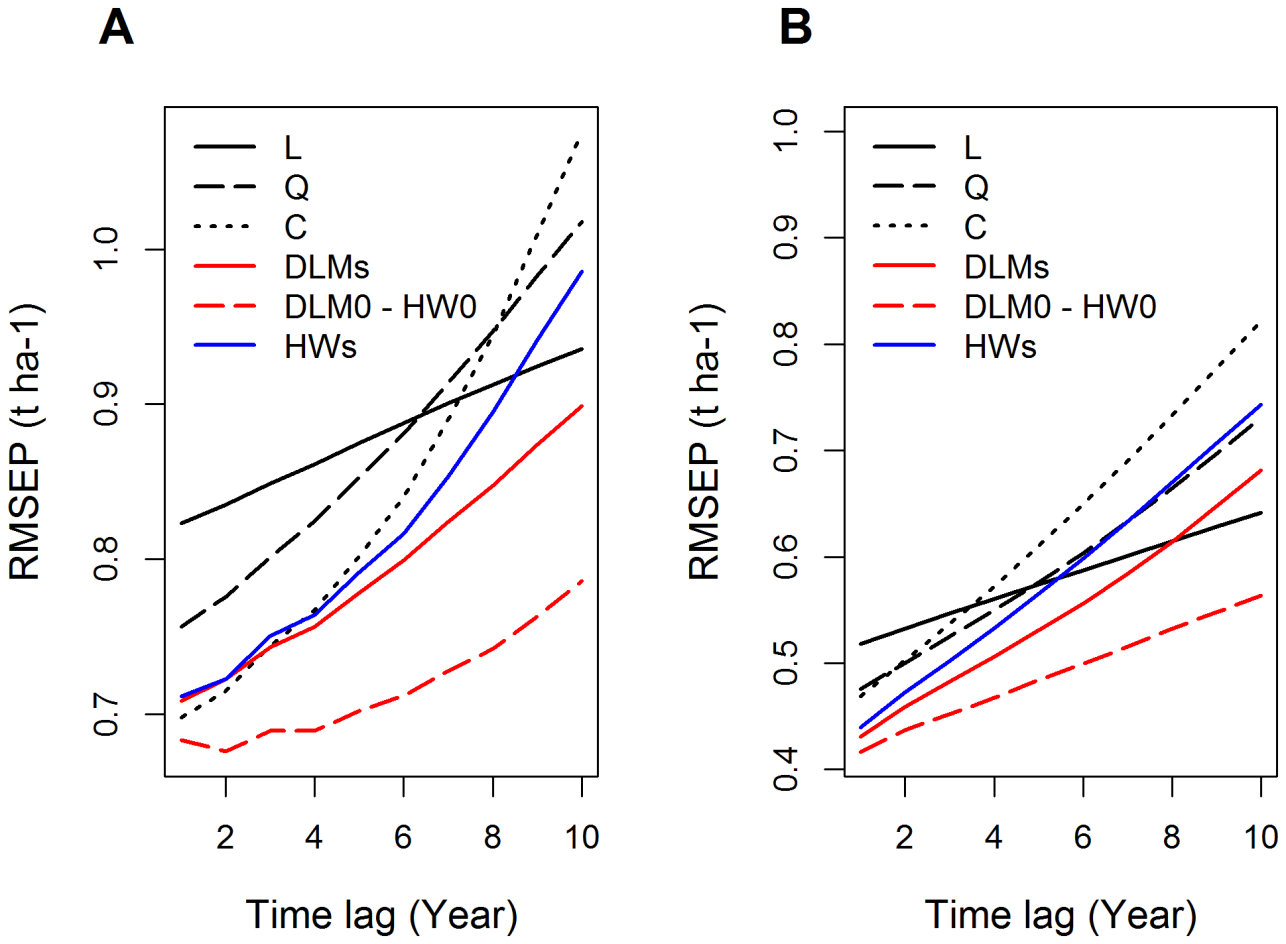
RMSEP of different statistical models as a function of time lag. A: France. B: Global.

In Figure 4, RMSEP values are displayed as a function of time lag (noted *k* in Eq.(12)) for the various models. RMSEP was found to increase as a function of time lag for all models. Short-term predictions were thus more accurate than long-term predictions, for all models. However, RMSEP did not increase at the same rate for all models. The rate of increase of RMSEP was greater for model C than for the other models, particularly model L. The RMSEP values of model C were close to those obtained for the best models for time lags of one or two (i.e., for predictions one-year ahead and two-year ahead), but the RMSEP values of model C were much higher than those of the best models when the time lag exceeded eight (Figure 4, Tables 2 and 3). Overall, greater differences in predictive performance between models were observed for long-term predictions than for short-term predictions (Figure 4).

**Table 2:** Root mean square error of one-year ahead predictions (RMSEP) obtained with two different datasets for several statistical models: linear regression (L), quadratic regression (Q), cubic regression (C), dynamic linear models with and without trend (DLMs, DLM0), and Holt-Winters with and without trend (HWs, HW0).

			Models						
Dataset	Quantity	Units	L	Q	C	DLMs	DLM0	HWs	HW0
France	RMSEP	t ha^−1^	0.82	0.76	0.70	0.71	0.68	0.71	0.68
	Difference^a^	%	18.73	10.49	1.77	3.40	0.09	3.98	0.00
Global	RMSEP	t ha^−1^	0.52	0.48	0.47	0.43	0.42	0.44	0.42
	Difference^a^	%	24.45	14.24	12.54	3.43	0.02	5.55	0.00

a The differences with respect to the lowest RMSEP values are expressed as a percentage of the lowest RMSEP values (RMSEP_min_); Difference = 100*(RMSEP – RMSEP_min_)/RMSEP_min_.

**Table 3:** Root mean square error of ten-year ahead predictions (RMSEP) obtained with two different datasets for several statistical models: linear regression (L), quadratic regression (Q), cubic regression (C), dynamic linear models with and without trend (DLMs, DLM0), and Holt-Winters with and without trend (HWs, HW0).

			Models						
Dataset	Quantity	Units	L	Q	C	DLMs	DLM0	HWs	HW0
France	RMSEP	t ha^−1^	0.94	1.02	1.08	0.90	0.79	0.99	0.79
	Difference^a^	%	19.02	29.52	36.90	14.35	0.00	25.42	0.08
Global	RMSEP	t ha^−1^	0.76	1.04	1.22	0.98	0.70	1.13	0.70
	Difference^a^	%	8.68	47.66	74.46	39.81	0.16	60.78	0.00

a The differences with respect to the lowest RMSEP values are expressed as a percentage of the lowest RMSEP values (RMSEP_min_); Difference = 100*(RMSEP – RMSEP_min_)/RMSEP_min_.

The models with the lowest RMSEP values were DLM0 and HW0, for all time lags tested. The RMSEP curves (Fig. 4) of these two models were visually indistinguishable, because the RMSEP values of DLM0 and HW0 were almost identical (Tables 2 and 3). For predictions 10-year ahead, the RMSEPs of DLM0 and HW0 for Datasets 1 and 2 were 74% and 37% lower, respectively, than the RMSEP of model C (Table 3).

DLMs was ranked third (after DLM0 and HW0) for most of the time lags considered, with a few exceptions. Several models (C, DLMs, and HWs) gave similar RMSEP values for Dataset 2 for time lags below 3 (Fig. 4A) and model L slightly outperformed model DLMs for Dataset 1 for time lags greater than eight. In all other cases, DLMs outperformed models C and HWs. In addition, DLMs was systematically better than model Q (Fig.4, Tables 2 and 3). Thus, although Q and DLMs included the same number of parameters (3), DLMs gave more accurate yield predictions.

Although yield predictions were more accurate in average with DLM0 than with DLMs, Table 4 shows that, compared to DLM0, the RMSEP of DLMs was lower in 21.7 to 32% of the considered geographical areas (*départements* or countries) depending on the dataset and the time lag. This result indicates that DLM0 was not systematically the best model for all geographical areas, and that DLMs performed better than DLM0 in about one-fifth to one-third of the geographical areas. The percentages of cases where DLMs was better than DLM0 were even higher when calculated over a restricted dataset including geographical areas characterized by a high yearly yield increase rate (e.g., Brazil); DLMs performed better than DLM0 in 30 to 50% of the geographical areas showing a strong yield increase rate in 2010, i.e. a yield increase rates higher than the median of the increase rates estimated with DLMs in 2010 (Table 4).

**Table 4:** Percentages of geographical areas (*départements* for the dataset France, and countries for the global dataset) where the model DLMs has a lower RMSEP than the model DLM0.

		% of cases where DLMs is more accurate than DLM0 over		
Dataset	Time lag (Year)^a^	all areas^b^	areas with low yield increase rates^b^	areas with high yield increase rates^b^
France	1	21.7%	13%	30.4%
France	10	32.6%	15.2%	50%
Global	1	28.4%	7.8%	49%
Global	10	29.4%	15.7%	43.1%

a RMSEP was computed for predictions one-year ahead and ten-year ahead.

b Percentages were computed over all geographical areas, over areas with low estimated increase rates (i.e., *départements*/countries showing an increase rate lower than the median of the increase rates estimated with DLMs in 2010), and over areas with high estimated increase rates (i.e., *départements*/countries showing an increase rate higher than the median of the increase rates estimated with DLMs in 2010).

## Discussion

### Model performance for estimating and predicting yields

DLM0 had the lowest RMSE and, thus, the best goodness-of-fit to past data. HW0 and DLM0 had the lowest RMSEP and gave the most accurate future yield predictions. As the RMSEP values obtained with the HW0 and DLM0 models were very similar for all the dataset × time lag combinations considered in this paper, it is difficult to choose between these two models for the prediction of future yields. The DLMs model was ranked second for RMSE and third for RMSEP in most cases. In addition, compared to DLM0, DLMs led to more accurate predictions in 30 to 50% of the geographical areas characterized by a strong yield increase rate (Table 4). The models with the least accurate predictions were the linear model L for short-term predictions, and the model C for long-term predictions (Fig. 4). HWs gave intermediate results.

This study did not cover all the existing techniques for analyzing time series. In particular, we did not consider ARMA models in this study because analyses of the residuals of regression models revealed no significant autocorrelation. The use of such models was, therefore, not really justified in this study. One limitation of this study is that it was not possible to assess the performances of the linear-plus-plateau model for all the available data, but we were able to calculate its RMSE values for a restricted dataset including data from 56 French *départements*. It was not possible to fit the LP model to the full dataset for France or to the global dataset, due to convergence issues. It was also not possible to calculate RMSEP values for this model. As many of our time series displayed no clear plateau, it was not possible to fit the LP model in many cases. However, the RMSE values obtained with the restricted France dataset showed that, when convergence was achieved, this model did not perform as well as DLM0, DLMs, and C (Table 1). The LP model is, therefore, probably not a good choice.

The performance of these models may be improved by including explanatory variables related to climate and to farmers’ practices. Prost et al. [23] used linear regression models to predict wheat yields in function of several variables such as sum of temperature, frequency of low temperatures, radiation, water balance, plant density, soil nitrogen, and disease severity. However, these authors showed that it was difficult to select the most relevant explanatory variables due to the lack of stability of the results of classical statistical selection procedures. Moreover, the use of such explanatory variables does not seem very suitable for predicting yields one or several years ahead because inputs related to climate and farmers’ practices are difficult to predict on the long term.

### Yield increase rate estimation

DLMs gave higher RMSE and RMSEP values than DLM0 and HW0. On the basis of these criteria, this model cannot therefore be considered the best choice. However, DLMs has an interesting practical advantage, in that it can be used to estimate both yield levels and yearly yield increase/decrease rates (noted 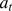 and 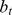 in Eq.(9)), whereas DLM0 and HW0 estimate only yield levels (i.e., only 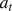). The estimation of yield increase rates is useful, because the values obtained indicate whether yield is stagnating, decreasing or increasing in the geographical areas of interest. When estimated dynamically every year, yield increase rates reveal changes in yield trends over time and provide useful information about trend changes. Yearly yield increase/decrease rate is an important parameter in foresight studies on food security, because it is used to determine whether food and feed supplies fit food and feed demands [8], [14]. According to Ye et al. [24], yield increase rate is a good indicator of food security. It is, therefore, useful to estimate this parameter from yield time series, especially when the yield increase rate is high.

Models L, Q, C, LP, HWs and DLMs can all be used to estimate yearly yield increase rates from yield time series. HW0 and DLM0 provide information about yield level, but not about yield increase rate. Of the models estimating yield increase rate, DLMs had the lowest RMSE and the lowest RMSEP values in almost all cases (Table 1, Figure 4). Based on these results, it seems logical to choose DLMs for the estimation of yield increase rates. Another advantage of DLMs is that it can estimate yearly yield increase rates for a great diversity of yield time series following very different trends. This is because DLMs describes yield dynamics by a stochastic process, without the requirement for a strong deterministic assumption. This is an important advantage for the estimation of yield increase rates and analysis of the dynamics of these rates. With DLMs, it is possible to determine whether yield is increasing, decreasing or stagnating, without assuming that yield follows any given analytical trend, such as a linear, quadratic, cubic or linear-plus-plateau pattern. Like DLMs, HWs makes no strong assumption about yield trend and is thus very flexible. However, the RMSEP values of HWs were systematically higher than those of DLMs (Figure 4) and HWs gave less accurate predictions. For these reasons, we believe that DLMs should be preferred for estimating yearly yield increase rates and their dynamics.

Our results demonstrate that dynamic linear models are powerful tools for analyzing yield time series. Surprisingly, this type of model has not yet been used for the analysis of yield time series. This is probably because the packages implementing dynamic linear models are recent [19]. Due to the availability of powerful packages such as dlm in R [20], this type of model will probably be more widely used in the future.

### Is wheat yield stagnating in France and around the world?

**Figure 5:**
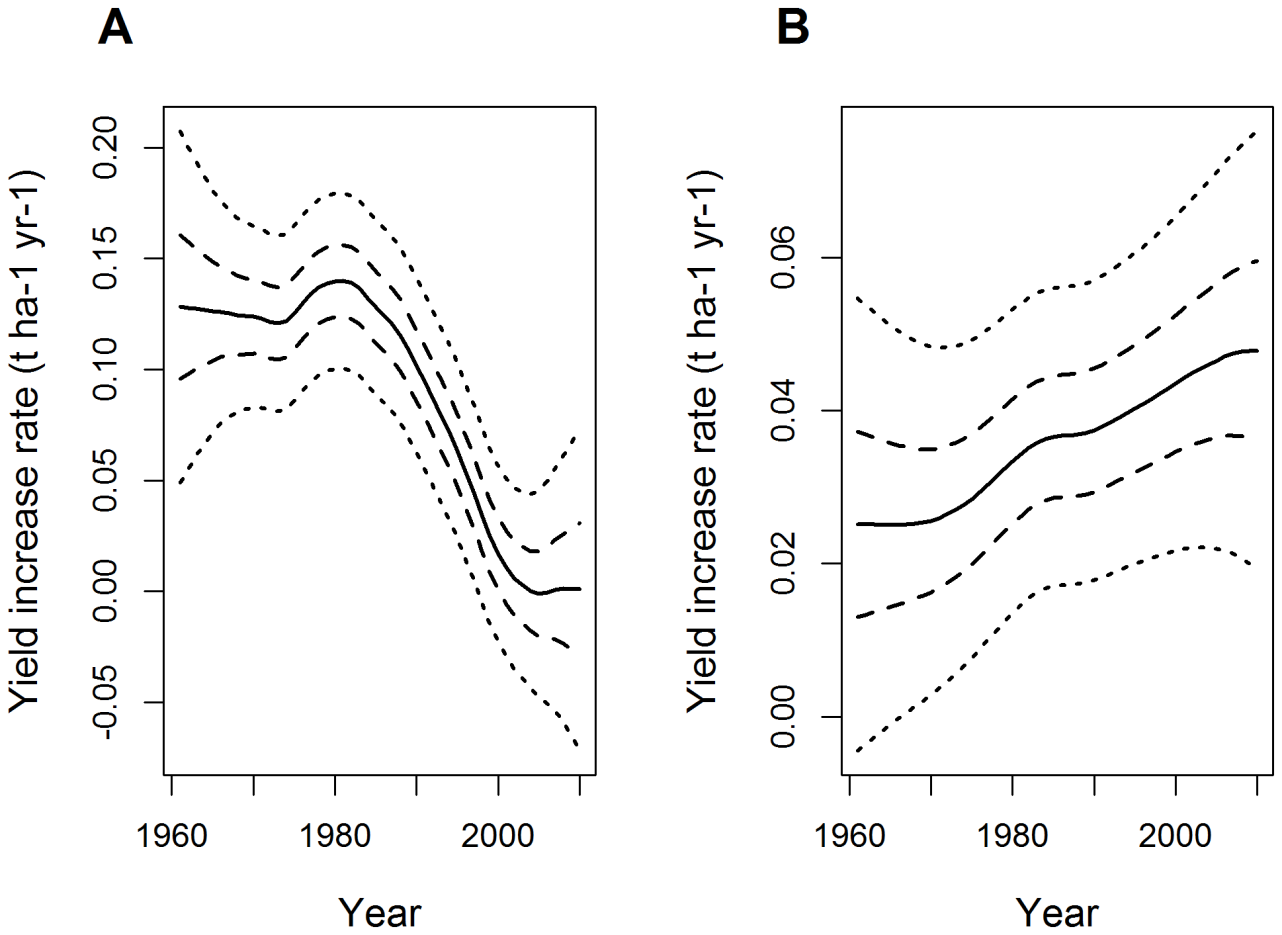
Estimated yield increase rates obtained with DLMs for wheat from 1960 to 2010 (continuous line), first and third quartiles (dashed lines), and 95% confidence intervals (dotted lines). A: France. B: Brazil.

**Figure 6:**
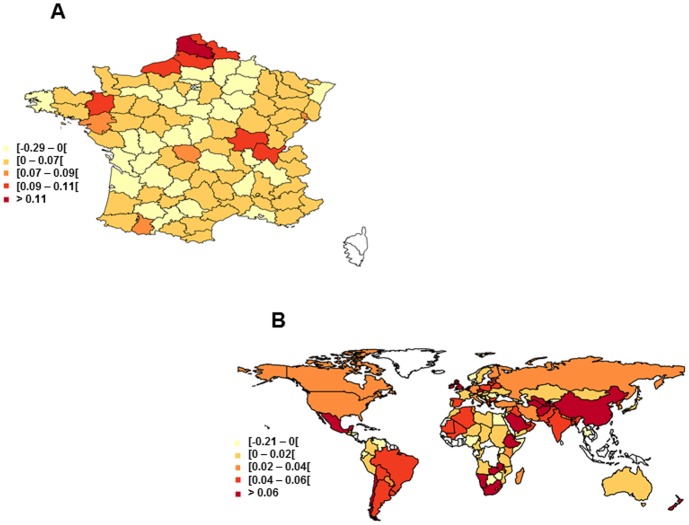
Estimated yield increase rates (t ha^−1^ year^−1^) obtained with DLMs for wheat. A: France in 2011. B: World in 2010. Countries for which wheat yield data are not available are indicated in white.

The practical value of DLMs is illustrated in Figures 5 and 6. Figure 5 shows yearly wheat yield increase rates estimated by the DLMs model for France and Brazil from 1961 to 2010. The 1^st^ and 3^rd^ quartiles, and the 95% confidence interval are displayed on the same figure to facilitate assessments of uncertainty. From the 1960s to the mid-1970s, the estimated increase in rate of yield was about 0.13 t ha^−1^ year^−1^ in France. This value increased slightly, from 1975 to 1981 and decreased steadily thereafter until 2005. The estimated rate of yield increase was about zero in France in 2010. The pattern of change in yield increase rate was very different in Brazil (Fig. 5B). In 1961, yield increase rate was about 0.025 t ha^−1^ year^−1^ and was, thus, much lower than that in France at the same time. However, yield increase rate steadily rose in Brazil from the 1970s to 2010, and was estimated at 0.045 t ha^−1^ year^−1^ in 2010. The confidence intervals reveal that the uncertainty associated with the estimated yield increase rate is high in both France and Brazil.

The maps in Figure 6 show wheat yearly yield increase rates estimated for French *départements* in 2011 and for the world in 2010. In France, the within-country variability of yield increase rate was high. Wheat yield was found to have stagnated in most French *départements*, but the yield increase rates estimated in 2011 ranged from negative values (yield decrease) to values of more than 0.11 t ha^−1^ year^−1^ (i.e., about as high as the highest yield increase rate recorded in France at national level, shown in Fig. 5A). Between-country variability was very high (Fig. 6B). Wheat yields were found to be stagnating or even declining in many countries (in France, but also in Norway, Sweden, Portugal, and several countries in Eastern Europe, Africa and South America), but estimated yield increase rates were above 0.06 t ha^−1^ year^−1^ in several countries in Europe, Asia, Africa and America.

**Figure 7:**
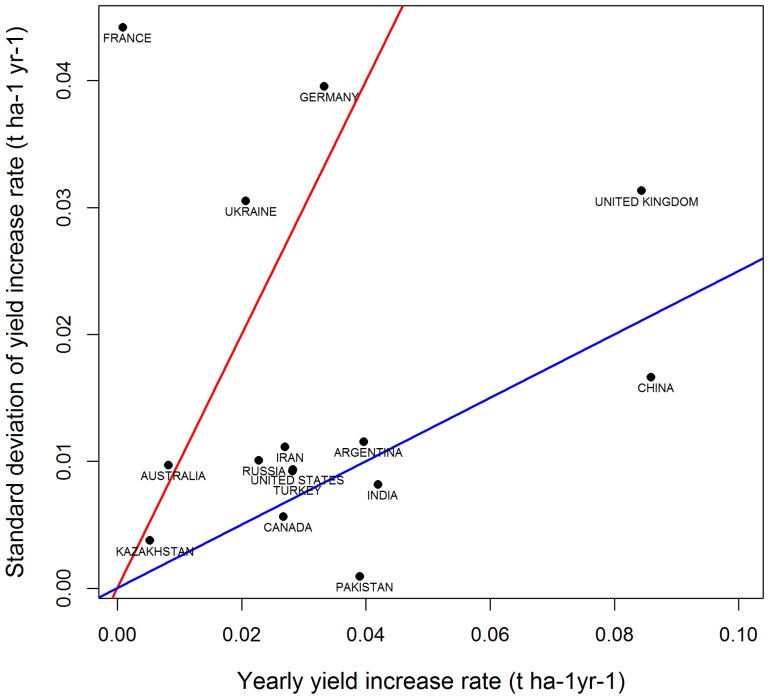
Standard deviation of yield increase rate (t ha^−1^ yr^−1^) *versus* estimated yield increase rate (t ha^−1^ yr^−1^) obtained with DLMs for the top 15 largest wheat producing countries. Countries above the red line and countries below the blue line show a coefficient of variation (100*standard deviation/estimated value) higher than 100% and lower than 25% respectively.

We will not discuss in detail here the reasons for these differences. However, it may be of interest to identify the factors potentially accounting for differences between the geographical areas in which yield is stagnating or decreasing and those in which it is continuing to increase. Three types of factor have been identified in previous studies; a slowing of genetic improvement, changes in agricultural practices and climate change. According to Brisson *et al*. [3] and Oury *et al.* [25], genetic improvement has not slowed down in the recent past and is therefore unlikely to account for yield stagnation in France. According to several recent studies, yield stagnation may partly reflect changes in agricultural practices and climate change [3], [11], [25], but there is currently no consensus in the scientific community about the causes of yield stagnation. In addition, Figure 7 shows that the uncertainty associated with the estimated yield increase rate is high for several major wheat producing countries. The coefficient of variation of the estimates was higher than 100% for four of the 15 top largest wheat-producing countries (Australia, France, Germany, Ukraine), and was lower than 25% for only four of these 15 countries. Due to this high uncertainty, the identification of explanatory variables is difficult. Yield increase rate values were found to differ considerably between the different regions in France (Figure 6), suggesting that efforts to identify the main causes of yield stagnation should focus at local, rather than national scale.
